# Enhancing water retention and mechanisms of citrus and soya bean dietary fibres in pre-fermented frozen dough

**DOI:** 10.1016/j.fochx.2024.101269

**Published:** 2024-03-05

**Authors:** Tianyu Xiao, Mingkun Sun, Shuwang Cao, Jianxiong Hao, Huan Rao, Dandan Zhao, Xueqiang Liu

**Affiliations:** a College of Food Science and Biology, Hebei University of Science and Technology, No.26 Yuxiang Street, Shijiazhuang, China; b Shijiazhuang Beirong Foods Co., Zhengding, Shijiazhuang, China

**Keywords:** Pre-fermentation, Water migration, Frozen dough, Citrus fibre, Soya dietary fibre

## Abstract

In recent years, the production of prepared and frozen foods has increased with economic development. However, during freezing, moisture migration forms ice crystals that damage food structure and reduce quality. This study investigates moisture migration changes in pre-fermented dough during frozen storage and effectiveness of Citrus fibre (CF) and Soya dietary fibre (SDF) on quality improvement. Pre-fermented frozen dough properties were evaluated at different freezing storage days with CF and SDF. Results showed frozen storage reduced water retention, converting deeply bound water to weakly bound and free water. Freezable water content increased significantly from 53% (fresh) to 56.95% (60d-control), forming disruptive ice crystals in gluten protein structure. SDF had superior water flow restriction compared to CF, preventing large ice crystal accumulation, enhancing water-holding capacity, and maintaining gluten protein structure. These findings lay a theoretical foundation for improving quality and industrial applications of pre-fermented frozen dough.

## Highlights

• Freezable water of pre-fermented frozen dough increased by 7.45% than fresh after 60 d.
• The deep-bound water of the dough with soybean fibre was 1.2 times that of control.
• Citrus fibre and soybean fibre can maintain gluten by reducing ice crystal formation.
• High crystallinity of soybean fibre enables it to have higher water retention.

## Graphical abstract

**Figure:**
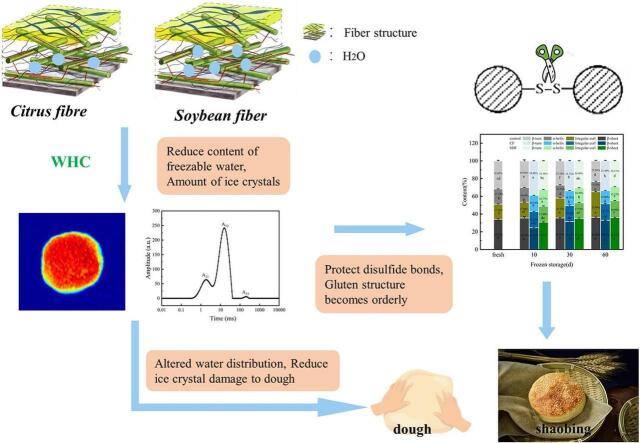

## 1 Introduction

Frozen dough is a new process of bread production developed in European countries since the 1950s, with many advantages such as time, labor and material savings, which is gradually becoming a mainstream trend in the bread industry, which has contributed to the rapid development of the chain business model in the baking industry. Pre-fermentation is a process in which the dough is treated by fermentation prior to production, a technique that significantly reduces the time required for the production of the finished product at a later stage and improves convenience for the chain. Overall, pre-fermented frozen dough has a clear advantage in terms of market share and growth rate in recent years. However, both the freezing process and the storage of frozen food can lead to a deterioration in the quality of the frozen food. On the one hand, the freezing process reduces the viability of the yeast and hurts the leavening power of the dough. On the other hand, water moves irreversibly from the “unfrozen” to the “frozen” state (B. B. Zhao et al., 2023). In this case, ice crystals form in the dough, destroying the gluten structure and reducing the quality of the dough (Ding et al., 2020).

The adverse effects of freezing on dough can be mitigated by the addition of food additives. These include hydrocolloids, anti-freeze proteins, emulsifiers, enzymes and dietary fibres. Hydrocolloids prevent water from freezing and maintain the rheological properties of the dough by competing with the starch in the dough for water. Antifreeze proteins prevent the growth of ice crystals, increase the resistance of the yeast to freezing and improve the physical properties of the dough (Xu, Huang, Wang, & Rayas-Duarte, 2009). The emulsifier is able to interact with the starch and prevent it from ageing, ultimately increasing the volume of the bread and reducing its hardness (Ribotta, Pérez, León, & Añón, 2004). The addition of bran fibre to the dough was found to release fermented dough gas, resulting in lower specific volume and harder bread (Ma et al., 2021). The addition of spinach flour to bread had richer nutritional properties, but reduced the sensory quality of the bread (Junejo et al., 2021). In conclusion, there are more studies on the sensory qualities of fibre-enriched dough and fewer studies on the role of fibre in the mechanism of water migration during frozen dough storage.

As the ‘7th nutrient’, dietary fibre can regulate blood sugar and blood pressure, weight control and the prevention of many cardiovascular diseases. The addition of appropriate amounts of dietary fibre to foods not only enhances the nutritional value of the food but also modifies the physic-chemical properties and the organoleptic quality of the food (Korus, Juszczak, Witczak, & Ziobro, 2020). Citrus fibre (CF) is a kind of dietary fibre extracted from natural citrus, which is not easy to be digested and absorbed by the human body, and has good water retention, oil retention and gel properties. Soybean dietary fibre (SDF) is characterised by its high yield and quality and can be used as a bulk raw material for the production of dietary fibre. It also has good water absorption and holding capacity, which can increase the moisture content of the dough, while the gel in the fibre can form a stable gel network with a three-dimensional structure, reducing the amount of starch regrowth and the rate of bread ageing. In terms of applied research on frozen dough in Chinese cuisine, there has been greater focus on steamed bread (Mantou) and frozen pre-baked dough of staple food. However, there has been relatively limited research conducted on freezing raw dough specifically for Shaobings (A traditional snack in northern China, which is made using wheat flour, water, and yeast as the primary ingredients). Therefore, this paper will study the effects of citrus fibre and soybean dietary fibre on water migration during the storage of pre-fermented frozen dough, as well as on shaobing, and analyze the structure of the two fibres to find out their mechanism of action.

In order to provide reference for the production process of pre-fermented frozen dough, differential scanning calorimetry, low field nuclear magnetic resonance and scanning electron microscopy were used to analyze and compare the effects of different dietary fibres on water migration of pre-fermented frozen dough.

## 2 Materials and methods

### 2.1 Materials

Wheat flour was obtained from Yihai Kerry Grain and Oil Industry Co., Ltd. (Shijiazhuang, Hebei, China), Active dry yeast was obtained from Angel Yeast Co. (Yichang, Hubei, China), Citrus fibre was obtained from LongRun Food (Qingdao, Shandong, China), Soybean dietary fibre was obtained from Shandong Jiahua Oil & Fat Co. (Liaocheng, Shandong, China). All chemicals were of analytical grade.

### 2.2 Preparation of pre-fermented frozen dough and frozen raw Shaobing

Preparation of pre-fermented frozen dough: Frozen dough included wheat flour of 300 g, active dry yeast of 2.4 g, 11.8 g sugar and deionized water of 182 mL (The dough should be supplemented with 1.5% CF or SDF based on the weight of wheat flour). A dough mixing machine (DL-TM018, Dongling Electric Co., Guangdong, China) was used to mix the above-mentioned ingredients. To make smooth dough with the optimum properties, 10 min machine mixing and 30 times of hand-kneading was needed. Subsequently, transfer the blended dough into a hermetically sealed incubator (BGZ-70, Boxun Medical Biology Co., Shanghai, China) set at 38 °C and allow it to ferment for a duration of 40 min. Finally, the dough is divided into 30 g small dough and put into the mold. After the quick-freezing, the dough is stored in the refrigerator at −25 °C.

Preparation of pre-fermented frozen raw Shaobing: make the dough of the above ratio into a rectangular sheet, it should then be evenly coated with a layer of shortening, using a 1:1 ratio of flour to vegetable oil. After that, fold and seal the dough before dividing it into small doses of 50 g each.

Baking conditions of frozen Shaobing: upper and lower heat 180 °C, 18 min, turn over at 9 min.

### 2.3 Analysis of moisture content in pre-fermented frozen dough

The moisture content of pre-fermented frozen dough was determined using the direct drying method. Approximately 1.5 g of dough was weighed and placed in an oven at 105 °C for a duration of 4 h. Subsequently, the weight of the dough was measured every 30 min until it reached a constant weight.

### 2.4 Freezable water content of pre-fermented frozen dough

The content of freeze able water in the dough was determined by using Differential scanning calorimeter (DSC 3500, Netzsch, Germany). To determine the freezeable water content of the dough sample, a precise weighing of 2–10 mg from the center of the dough is conducted. The weighed sample is then carefully sealed in a crucible, while a blank crucible serves as a control. The assay procedure begins by maintaining a stable temperature of 25 °C for a duration of 5 min. Subsequently, the sample is gradually cooled down to −30 °C at a controlled rate of 10 °C /min. It is kept at −30 °C for an additional 5 min before being slowly heated back up to 20 °C at a rate of 10 °C/min. The Proteus Analysis software is utilized to calculate the melting enthalpy (ΔH) of the sample. Finally, the freezeable water content is determined using the following formula:

$$\mathrm{Fw}=\frac{\mathrm{\Delta H}}{\mathrm{Wt}\times H_{0}}\times 100\%$$

Where Fw represents the freezable water content (%) of the sample, Δh represents the melting enthalpy (J/g) of the sample, H0 represents the melting enthalpy of ice formed by pure water per unit mass, and 333 J/g and Wt (%) represent the water content of the sample.

### 2.5 Moisture distribution determination of pre-fermented frozen dough

The moisture distribution of the frozen dough was determined by referring to the Ren (Ren et al., 2016) method using a low field nuclear magnetic resonance apparatus (LF-NMR, MESOMR23-060H-I, Suzhou Niumai Electronic Technology Co., Suzhou, China). After thawing (38 °C, 30 min) the frozen dough was accurately weighed at the center 5.00 ± 0.01 g in a special glass tube for NMR, and the moisture distribution was determined using a low-field NMR instrument. Please refer to the supplementary materials for detailed conditions (Table s-1).

### 2.6 Nuclear magnetic imaging analysis of pre-fermented frozen dough

To assess the dough's freezing characteristics, an accurate weighing of 5.00 ± 0.01 g was performed on samples taken from the center. A low-field NMR instrument was employed to collect information on the dough samples. Each sample was positioned at the center of a radio frequency coil within a permanent magnetic field. Data acquisition is carried out using specific pulse sequences that allow detailed analysis of the properties of the dough in different frozen storage. Please refer to the supplementary materials for detailed conditions (Table s-2).

### 2.7 Ice crystal form of pre-fermented frozen dough

The microstructure of the dough was observed using a scanning electron microscope (SEM, S4800). Firstly, the freeze-dried dough was cut into small pieces and fixed onto the sample stage, and then gold was sprayed on its surface. Finally, under an accelerating voltage of 5.0 kV, with a × 500 magnification, the samples were observed and photographed.

### 2.8 Fourier transform infrared spectroscopy (FTIR) of pre-fermented frozen dough

The content of protein secondary structure in dough was determined using FTIR (IRSpirit, Japan). The freeze-dried dough was ground and sieved through an 80-mesh sieve, and the dough powder and KBr were mixed in a ratio of 1:100, mixed and ground in an agate mortar under infrared light until powdered, and pressed into thin slices for the determination. The test parameters were: spectral range 400–4000 cm^−1^, resolution 4 cm^−1^, and number of scans 32.

### 2.9 Analysis of free-SH in pre-fermented frozen dough

The lyophilized dough powder sample was accurately weighed at 50 mg. Subsequently, 5 mL of SDS-TGE solution and 100 μL of DTNB-TGE solution were added. The mixture was vigorously shaken during the vortex shaking period to ensure complete dissolution of the solids. Afterward, the reaction was allowed to proceed for 20 min at room temperature, shielded from light. Following this, the sample was centrifuged at 10,000 rpm for 20 min. The absorbance value of the resulting supernatant at 412 nm was then determined. In order to establish a standard curve, the glutathione standard was utilized.

### 2.10 Gluten structure of pre-fermented frozen dough

Laser Confocal Scanning Microscope (CLSM, Leica Co., Germany) was used to observe the structure of gluten proteins in frozen dough, and the samples were fixed on a sample tray using Lycra adhesive, cut into 20 μm slices, stained with a mixture of fluorescent dye rhodamine B (0.025%, *w*/*v*) and fluorescein isothiocyanate FITC (0.25%, *w*/w) (1:1) for 1 min, and rinsed using deionized water. The detection wavelengths were 575 nm–620 nm for rhodamine B and 488 nm–518 nm for FITC, and the magnification was 200×, and the resolution of the digital image files was 1024 × 1024 pixels.

### 2.11 Dynamic rheological properties of pre-fermented frozen dough

Internal (0.5 ± 0.05) g of spherical dough was weighed and the elastic modulus G′ and viscous modulus G″ were determined using a dynamic rheometer. A P35 probe was utilized, with a set temperature of 25 °C, a frequency scanning range of 0.1–10 Hz and a gap of 1 mm.

### 2.12 Texture properties of pre-fermented frozen dough

Texture properties of pre-fermented frozen dough and shaobing were measured by Texture analyzer (TA. XT Plus, Stable Micro System Co., Britain). Texture properties of pre-fermented frozen dough using a 1-in. diameter spherical probe, please refer to the supplementary materials for detailed conditions (Table s-3). Texture characteristics of pre-fermented frozen green shaobing using p5 probe, please refer to the supplementary materials for detailed conditions (Table s-4).

### 2.13 Determination of fibres by FTIR

FTIR spectra of fibres were performed using FTIR spectrometer (IRSpirit, Japan) according to the method reported by Lu (Lu & Zhu, 2023). with some modifications. Fibre sample was homogeneous mixed with KBr at a ratio of 1:100 (*w*/w, dry basis). Powder sample was pressed into transparent sheet and scanned across the wavelength interval of 4000–400 cm^−1^ at a resolution of 4 cm^−1^ by 10 scans. The background was KBr.

### 2.14 Determination of fibres by X-ray diffraction (XRD)

The crystallinity of the fibres was conducted by XRD (D/MAX-2500, Japan). The measurement conditions: scanning frequency 4(°)/min, incidence Angle 5° ∼ 40°, step size 0.06°, using software Jade 6.0 to calculate the relative crystallinity.

### 2.15 Determination of water-holding capacity (WHC) of fibres

The 1 g fibre sample was put into a centrifuge tube and weighed. Add 30 mL distilled water and mix well, place for 1 h, centrifuge at 3500 r/min for 30 min, pour off the supernatant and weigh. The WHC is the difference between the masses of two centrifugal tubes.

### 2.16 Determination of gelatinization characteristics of wheat flour with fibre

Rapid Visco-Analyzer (RVA) was used for the determination. Wheat flour with fibre was corrected with 13% wet basis. 3.5 g wheat flour with fibre and 25 mL deionized water were weighed into an RVA aluminum box and put into the RVA for determination according to the set test procedure.

### 2.17 Determination of the powdery properties of wheat flour with fibre

Chopinp+ standard scheme mode was selected, water content and estimated water absorption of each group were set, the mass of dough formed with fibre wheat flour and water was set to 75 g, and the addition mass of fibre wheat flour and water was adjusted, so that the target torque C1 was within (1.1 ± 0.05) N·m.

### 2.18 Statistical analysis

All analyses in this study were performed in at least triplicate. Significance analyses were performed using SPSS 26 to verify significant differences (*p* < 0.05). Graphs were made using origin 2018 for the experimental results.

## 3 Results

### 3.1 Effect of CF and SDF on moisture properties of pre-fermented frozen dough

#### 3.1.1 Freezable water for pre-fermented frozen dough

The water in dough can be divided from its frozen state into freezable water and non-freezable water. The former consists of free water and weakly bound water that can easily flow and freeze, while the latter consists of bound water tightly bound to proteins and starches that cannot easily flow and freeze (Kerch, Glonin, Zicans, & Meri, 2012). During the freezing and storage of dough, freezable water will damage the gluten protein network structure by promoting ice crystallization and recrystallisation of ice, thus compromising the quality of the dough. In this study, DSC was applied to assess the effect of CF and SDF on the freezable water content in doughs. The enthalpy of melting (ΔH) is the key parameter obtained from the DSC test and the freezable water content can be calculated according to Eq.

The freezable water content of pre-fermented frozen dough in different frozen storage was shown in Table 1. After 10, 30 and 60 days of freezing and storage, the ΔH of the control pre-fermented frozen dough increased significantly from 79.24 J/g fresh to 81.27 J/g, 82.48 J/g and 83.22 J/g (*p* < 0.05) and the freezable water content increased from 53.00% of fresh to 54.88%, 55.99% and 56.95% respectively. This phenomenon may be due to the fact that freezing weakened the protein-water forces, allowing more bound water to be released from the dough (Bot, 2003).

**Table 1:** Effect of CF and SDF on the freezable water content of pre-fermented frozen doughs.

Frozen storage(d)	group	ΔH(J/g)	water content(%)	Freezable water(%)
fresh	control	79.24 ± 0.12^c^	44.89 ± 0.35^abc^	53.00 ± 0.22^d^
10	control	81.27 ± 0.18^b^	44.47 ± 0.28^bcd^	54.88 ± 0.12^c^
	CF	78.45 ± 0.12^cd^	45.24 ± 0.52^a^	52.07 ± 0.16^e^
	SDF	76.90 ± 0.56^e^	45.39 ± 0.52^a^	50.87 ± 0.53^f^
30	control	82.48 ± 0.96^ab^	44.24 ± 0.00^cd^	55.99 ± 0.92^b^
	CF	79.35 ± 0.22^c^	45.00 ± 0.22^ab^	52.94 ± 0.29^d^
	SDF	77.66 ± 0.28^de^	45.21 ± 0.21^a^	51.58 ± 0.37^ef^
60	control	83.22 ± 0.00^a^	43.88 ± 0.21^d^	56.95 ± 0.00^a^
	CF	79.21 ± 0.61^c^	44.73 ± 0.14^abc^	53.18 ± 0.45^d^
	SDF	77.42 ± 0.58^de^	45.01 ± 0.01^ab^	51.59 ± 0.38^ef^

Means with different letters for each column indicate a significant difference at *P* < 0.05, results are shown as mean ± standard deviation. CF and SDF represent frozen dough containing citrus fibre and frozen dough containing soybean fibre, respectively.

The addition of CF and SDF significantly reduced the content of freezable water in pre-fermented frozen dough and delayed its increase during frozen storage. After 60 days of frozen storage, the freezable water content was found to be 53.18% and 51.59% for pre-fermented frozen dough with CF and SDF added, respectively. These findings demonstrated the effectiveness of CF and SDF in inhibiting the transition of pre-fermented frozen dough from non-freezable water to freezable water. However, when comparing the CF group with the SDF group (Table 1.), it can be observed that non-freezable water in the dough with CF during frozen storage underwent more transformation into freezable water. Therefore, it can be concluded that the inhibitory effect of CF on the transformation of non-freezable water into freezable water is lower compared to SDF.

#### 3.1.2 Water mobility of pre-fermented frozen doughs

**Fig. 1:**
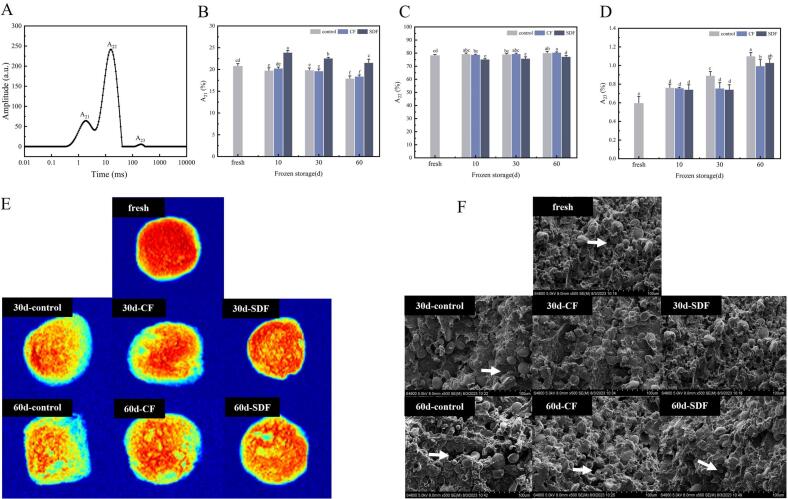
Effects of CF and SDF on moisture characteristics of pre-fermented frozen dough. A show the T2 spectrum of LF-NMR. B, C and D are the effects of CF and SDF on the content of A_21_, A_22_ and A_23_ of pre-fermented frozen dough, respectively. E Show nuclear magnetic imaging of pre-fermented frozen dough (Red indicates areas of high moisture; dark blue indicates anhydrous backgrounds). F Show SEM images of pre-fermented frozen dough (The white arrows show holes caused by ice crystals during frozen storage). (For interpretation of the references to color in this figure legend, the reader is referred to the web version of this article.)

Low field nuclear magnetic resonance (LF-NMR) has been widely used to study the state, distribution, composition, and migration patterns of water in food. A_21_, A_22_, and A_23_ represent the ratios of peak areas corresponding to deeply bound water, weakly bound water, and free water, respectively. As shown in Figs. 1B, C, and D, the relative content of weakly bound water in the dough was found to be the highest, ranging from 75.33% to 80.26%. This was followed by deeply bound water, ranging from 17.79% to 23.93%, and finally free water, ranging from 0.60% to 1.10%.

After 60 days of storage, the A_21_ content of pre-fermented frozen dough without fibre significantly decreased from 20.87% to 17.97% (*P* < 0.05). In comparison to fresh dough, A_23_ increased by 83%, while A_22_ showed a slight improvement but not a significant change. These results indicate that deeply bound water in the pre-fermented frozen dough gradually transforms into weakly bound water and free water during frozen storage, consequently altering the water distribution in the dough.

The variation of water content in the pre-fermented frozen dough with CF and SDF was similar to that in the control group. However, it is worth noting that the content of deeply bound water in the SDF group remained higher than that in the control group over the course of 60 days. On the other hand, the contents of A_21_, A_22_, and A_23_ in the pre-fermented frozen dough with CF added were nearly identical to those in the control group under the same frozen storage conditions. These observations suggest that CF is less effective than SDF in altering the moisture distribution in pre-fermented frozen dough.

#### 3.1.3 Internal moisture distribution and physicochemical changes

In this study, MRI was used to evaluate changes in the water signal of pre-fermented frozen dough during storage (Fig. 1E). MRI pseudo-color images with a uniform color area corresponded to the dough matrix with even water distribution, while different colors or dark areas corresponded to the moisture gradients or bubbles induced by the deformation of gluten network and water migration. Color changes from red to yellow to light (deep) blue indicated the gradual decrease in the proton density (Li, Ma, Zhu, Guo, & Zhou, 2017).

The structure and water distribution of the fresh pre-fermented dough were homogeneous in the red color. After 30 days of frozen storage, the moisture distribution of the dough becomes uneven (with different colors), with the inner red color weakening and the edges appearing blue. Subsequently, after 60 days of frozen storage, the size and number of blue cells in the dough significantly increased, while the internal redness decreased. This confirms that freezing weakens the water retention ability of the pre-fermented dough. Comparing it with the dough without fibre addition, the pre-leavened dough with fibre addition exhibited a higher water content and more uniform water distribution after 60 days of low-temperature storage. This reflects the presence of different structures in the fibre, each with varying abilities to interact with water. The dough containing SDF is more evenly distributed and has a higher water content than the dough with CF. This difference may be attributed to SDF with higher water-holding capacity, which reduces water flow and alters its distribution.

#### 3.1.4 Ice crystal morphology analysis

The morphological characteristics of ice crystals in pre-fermented frozen dough can be observed by scanning electron microscopy. When ice crystals were present in the frozen dough, irregular holes could be observed in the micrographs at the same freezing storage time. The extent of damage to the dough structure can be assessed by observing the void formed by ice sublimation, which can reflect the distribution of ice formed in the mass sample before freeze-drying (Ding et al., 2020).

As seen in Fig. 1F, ice crystals had not formed in the fresh doughs. The gluten network structure was relatively continuous and tight, with complete/contact starch granules filling the gluten network, providing support to the dough's structure. After frozen storage, it was observed that the pores in the gluten matrix of the non-fibre pre-fermented dough were large and uneven, with most of the starch particles exposed to the gluten matrix. Consequently, the gluten network became looser and thinner. On the other hand, the dough with added fibre exhibited smaller and fewer pores after freezing, with a relatively continuous and dense gluten network. Furthermore, the starch particles were less exposed, particularly when SDF was added. These findings suggest that the addition of both CF and SDF can reduce the formation of ice crystals to some extent, thereby protecting the gluten structure from mechanical damage.

### 3.2 Effect of CF and SDF on protein properties of pre-fermented frozen dough

#### 3.2.1 Free SH of pre-fermented frozen dough

The disulfide bond can reinforce protein macromolecules, the formation of a strong network structure and the maintenance of structural stability. However, ice crystals formed during frozen storage break off -SS, increase SH content and disrupt the protein network structure. For this reason, the effect of freezing on the gluten network is usually assessed by measuring the free SH content (Shu, Wei, Liu, Liu, & Chen, 2022; L. Zhao et al., 2013).

**Fig. 2:**
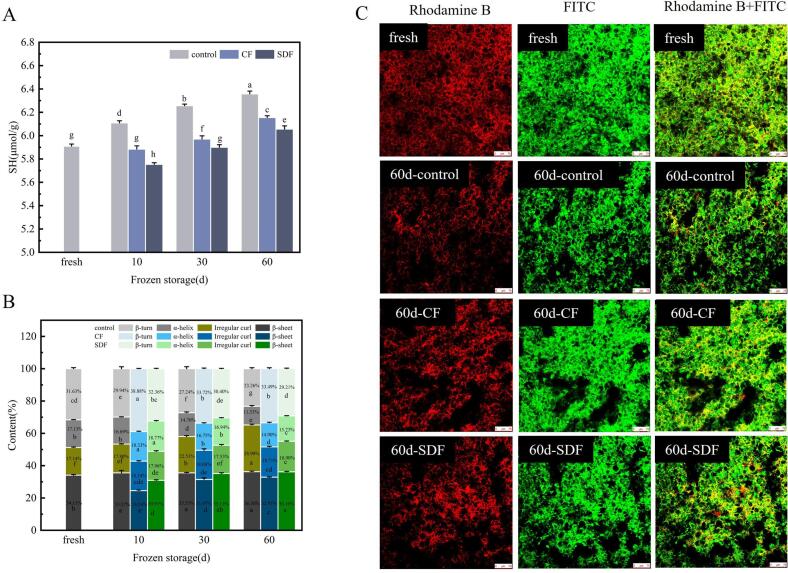
Effects of CF and SDF on the characteristics of gluten protein in pre-fermented frozen dough. A show the effects of CF and SDF on the free SH content of pre-fermented frozen dough. B shows the effect of CF and SDF on the secondary structure of proteins in pre-fermented frozen dough. C Shows CLSM images of pre-fermented frozen dough.

Fig. 2A demonstrates an increase in free sulfhydryl content as the frozen storage time increased. The fresh samples exhibited a free sulfhydryl content of 5.91 μmol/g. However, after 60 days of frozen storage, the samples without fibres showed an increased content of free sulfhydryl groups, reaching 6.36 μmol/g. On the other hand, samples with added CF and SDF had a free sulfhydryl content of 6.15 μmol/g and 6.06 μmol/g, respectively, which were significantly lower than the samples without fibres. This observation suggests that CF and SDF have the ability to reduce the increase in free SH content by protecting the network structure.

#### 3.2.2 Protein secondary structure of pre-fermented frozen dough

The changes in the secondary structure of pre-fermented dough during frozen storage were studied using Fourier transform infrared spectroscopy (FTIR), specifically analyzing the amide I region (1600–1700 cm^−1^) for detailed examination. Fig. 2B presents a summary of the corresponding results for the secondary structure features, expressed as a percentage of the total area of the amide I band spectrum.

A comprehensive analysis of the FTIR study results revealed that the α-helix content in the secondary structure of the pre-fermented frozen dough gradually decreased with an increase in freezing time, while the random coil content in the secondary structure gradually increased during frozen storage. After 60 days of frozen storage, the unadded fibre dough exhibited a decrease in α-helix content from 17.13% to 11.53%, with the random coil content reaching 28.90%. Similarly, after 60 days of storage, CF and SDF displayed α-helix contents of 14.90% and 15.73%, respectively, with significantly lower random coil contents compared to the dough without fibre.

The α-helix represents an ordered structure, while the random coil indicates a disordered structure. These findings suggest that frozen storage leads to a more disordered protein secondary structure in the dough. However, the presence of CF and SDF may protect the protein conformation by reducing the size and number of ice crystals formed within the dough.

#### 3.2.3 Microstructure of pre-fermented frozen dough

The microstructure of different frozen stored pre-leavened dough was observed by laser confocal microscopy, and the results were shown in Fig. 2C. At specific excitation wavelengths, the gluten network and starch particles appear red and green, respectively, when combined with Rhodamine B and FITC dyes, and yellow when the two macromolecules are cross-linked (Jia et al., 2019).

Wheat flour dough is considered a complex mixture comprising starch granules fully embedded in the gluten matrix, forming a continuous three-dimensional network. As the freezing storage time prolongs, changes occur in the distribution of starch particles around the gluten skeleton. Initially, the starch particles are uniformly distributed (fresh dough). However, after 60 days of storage (60d-control), the starch particles aggregate internally, resulting in a non-uniform distribution and the formation of large pores in the gluten network structure.

Comparatively, dough samples subjected to freezing storage for 60 days with added CF (60d-CF) and SDF (60d-SDF) exhibit a more pronounced network structure, with starch particles distributed on the gluten. These observations suggest that CF and SDF contribute to maintaining a more organized gluten network during frozen storage.

### 3.3 Effects of CF and SDF on physical properties of pre-fermented frozen dough

#### 3.3.1 Dynamic rheological properties of pre-fermented frozen dough

The dynamic rheology of dough is closely related to the processing and final quality of the product, three important indicators of dynamic rheological characteristics: elastic modulus (G′) represents the elastic nature of the substance; viscous modulus (G″) represents the viscous characteristics of the substance; the loss angle (tanδ) (tanδ = G”/G') reacts to the ratio of the system's viscoelasticity (Ye, Han, Zhao, Cao, & Tang, 2013).

**Fig. 3:**
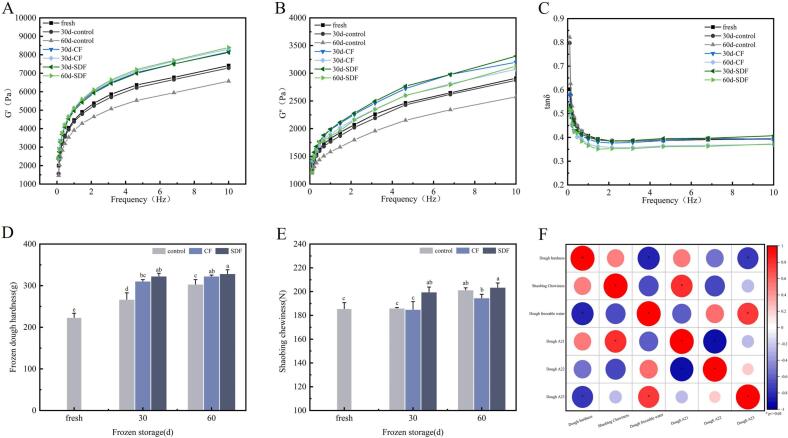
Effects of CF and SDF on physical characteristics of pre-fermented frozen dough. A, B and C show the effects of CF and SDF on G', G “and tanδ of pre-fermented frozen dough, respectively. D shows the effects of CF and SDF on the hardness of pre-fermented frozen dough. E shows the effects of CF and SDF on the chewiness of pre-fermented frozen raw Shaobings. F Shows the correlation between moisture properties of pre-fermented frozen dough and its finished product.

Fig. 3A, B and C show the rheological properties of pre-fermented frozen dough during frozen storage. In the frequency range of 0.1–10 Hz, both G' and G“ of dough increase with the increase of frequency, and G' of dough is always greater than G", indicating that the dough system is still viscoelastic, which is consistent with known reports (Shu et al., 2022). As can be seen from the figure, G' and G“ decrease with an increase in frozen storage time for both unadded and added fibre dough. The addition of CF and SDF increased the G' and G" of the dough. The change amount was small during the frozen storage process, which resulted in more stable dynamic rheological properties of the pre-fermented frozen dough. The elasticity of all dough samples was higher than their viscosity. After 60 days of frozen storage, the SDF group exhibited the lowest tan(δ). This may be attributed to the high degree of polymerization of proteins and fibres in the added SDF.

#### 3.3.2 Texture characteristics of pre-fermented frozen dough and Shaobing

Textural properties can directly affect the final quality and flavor of processed doughs. Fig. 3D illustrates the hardness of the pre-fermented frozen dough. During frozen storage, the inside of the dough loses water through the sublimation of ice crystals, resulting in an increase in the hardness of the dough, which in turn increases the hardness of the baked shaobing.

As the frozen storage period increases, the hardness of the dough in all groups also increases. This effect is particularly significant in the non-fibre group, where the hardness increases from 223.51 g (fresh) to 303.64 g (60d-control). Regardless of whether CF or SDF is added, the addition of fibre results in a greater hardness compared to dough without fibre. Furthermore, the highest hardness is observed when SDF is added. However, it should be noted that the addition of SDF does not significantly change the hardness of the dough during the 60-day storage period.

Fig. 3E illustrates that the chewiness of Shaobing did not increase significantly in the group without added fibre within the first 30 days of frozen storage. However, it increased significantly between 30 and 60 days of storage. The chewiness of Shaobing with CF was almost the same as that without CF. On the other hand, the chewiness of Shaobing in the SDF group was significantly higher than that in the group without SDF within the initial 30 days of storage, but the change was not significant within 60 days.

Fig. 3F displays the correlation analysis of the main parameters. The content of freezable water, A_21_, A_22_, and A_23_ of the dough were selected for correlation analysis with dough hardness and shaobing chewiness. The freezable water content and A_23_(free water) content of pre-fermented frozen dough were negatively correlated with dough hardness, respectively, while the A_21_(deeply bound water) content was positively correlated with shaobing chewiness. The addition of CF and SDF will significantly reduce the content of freezable water and increase the content of A_21_ (deep bound water), so the hardness of dough and the chewiness of shaobing will also increase according to the correlation.

### 3.4 Mechanisms behind variations in water retention of different fibres

#### 3.4.1 Fourier spectrograms of fibres

Through the overall comparison, it is found that CF and SDF have roughly the same peak structure. In principle, both are polysaccharides, consisting mainly of the water-soluble pectin and the water-insoluble cellulose, hemicellulose, lignin and so on. Pectin is a heteropolysaccharide linked by α-(1,4)-glucose linkages of galacturonic acid residues. Cellulose is a molecular substance linked by β-(1,4)-glucose linkages of d-glucose.

**Fig. 4:**
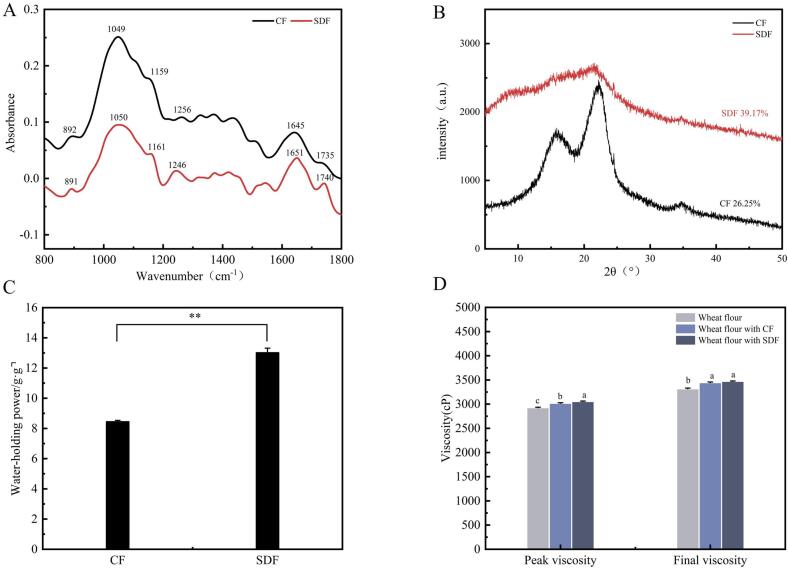
Analysis of structural characteristics of CF and SDF. A Shows the FTIR spectra of CF and SDF. B Show the XRD patterns of CF and SDF. C shows the WHC of CF and SDF. D Shows gelatinization characteristics of CF and SDF.

As shown in Fig. 4A, the absorption peak at 890 cm^−1^ belongs to cellulose and other β-glycosidic bonds, while the functional groups represented by the absorption peak at 1250 cm^−1^ are mainly dimensional alcohol and ester bonds characterizing the macromolecular structure of hemicelluloses. The obvious peak near 1735 cm^−1^ indicates that CF and SDF contain hypomethylated pectin (Y. Liu, Liu, Ibrahim, Yang, & Huang, 2018; Sivam, Sun-Waterhouse, Perera, & Waterhouse, 2013), and the difference in peak intensity here is likely to be the main reason for the different effects of CF and SDF.

#### 3.4.2 XRD analysis of fibres

As shown in Fig. 4B, CF and SDF exhibit distinct peaks at 2θ angles of 22° and 34°, respectively. These peaks are characteristic of cellulose type I, indicating that the fibres possess a crystalline structure with both crystalline and amorphous regions. The crystallinity levels of CF and SDF follow the order of SDF (39.17%) > CF (26.25%), indicating that SDF exhibits higher crystallinity compared to CF. This high crystallinity suggests stronger intracrystalline binding forces, robust hydrogen bonding within and between chains, and denser binding. XRD analysis further confirms that SDF possesses the most stable structure, followed by CF.

#### 3.4.3 WHC analysis of fibres

The hydrophilic groups present in dietary fibre, along with the porous matrix structure formed by polysaccharide chains, contribute to the substantial water retention capabilities. Water Holding Capacity (WHC) refers to the ability of a specific amount of dietary fibre to bind water under the influence of gravity or atmospheric pressure. Thus, WHC serves as a significant indicator reflecting the hydration properties of dietary fibre. In Fig. 4C, the disparity between CF and SDF holding capacities is depicted. The WHC of CF measures 8.45 g/g, whereas the WHC of SDF amounts to 13.02 g/g. Notably, the WHC of SDF is 1.54 times higher than that of CF. This observation aligns with the XRD findings discussed earlier, which indicated a positive correlation between fibre crystallinity and water holding capacity.

#### 3.4.4 Gelatinization characteristics of wheat flour with fibre

Fig. 4D showed that compared with wheat flour, the peak viscosity and final viscosity of wheat flour with CF and wheat flour with SDF are significantly increased. The peak viscosity is related to the expansion degree of the particles during heating. On the one hand, the pectin in the fibre has high viscosity after water absorption and expansion. On the other hand, the fibre and starch particles absorb water and expand together, and the stable fibre structure may accelerate the breakdown of starch particles and the leaching of starch molecules during the heating process, which synergically leads to higher peak viscosity.

#### 3.4.5 Powdery characteristics analysis of wheat flour with fibre

Table 2 presented the effects of CF and SDF on various Mixolab indicators related to the powdery characteristics of wheat flour. The water absorption of wheat flour is 60.6%, which can be increased to 62.4% with CF and 63.4% with SDF, indicating that SDF has a greater ability to improve water absorption in the dough compared to CF. The increase in the C1 value signifies enhanced water absorption in the dough, while C2, C3, C4, and C5 represent protein weakening, starch gelatinization rate, starch gelatinization stability, and starch recovery rate, respectively. As shown in Table 2, CF improves the protein weakening effect, starch gelatinization rate, and starch gelatinization stability of the dough. However, SDF does not have a significant impact on these parameters. Nevertheless, SDF reduces the C5 value, indicating a decrease in starch recovery rate and suggesting a delay in dough ageing (Tebben, Shen, & Li, 2018).

**Table 2:** Effects of CF and SDF on the characteristics of wheat flour.

Group	Water absorption /%	C1 /(N·m)	C2 /(N·m)	C3 /(N·m)	C4 /(N·m)	C5 /(N·m)
Wheat flour	60.6	1.090 ± 0.001^b^	0.549 ± 0.008^c^	1.903 ± 0.001^b^	1.794 ± 0.046^b^	2.799 ± 0.105^a^
Wheat flour with CF	62.4	1.111 ± 0.004^a^	0.621 ± 0.002^a^	2.006 ± 0.018^a^	1.914 ± 0.020^a^	2.758 ± 0.016^ab^
Wheat flour with SDF	63.4	1.121 ± 0.010^a^	0.566 ± 0.001^b^	1.896 ± 0.013^b^	1.757 ± 0.008^b^	2.568 ± 0.020^b^

Means with different letters for each column indicate a significant difference at *P* < 0.05, results are shown as mean ± standard deviation. C1, C2, C3, C4 and C5 represent water absorption, protein weakening, starch gelatinization rate, starch gelatinization stability and starch recovery rate, respectively.

## 4 Discussion

Many studies on frozen dough storage have focused on the impact of water migration as a significant factor in reducing the quality of frozen dough products. During the freezing process, water irreversibly migrates from an “unfreezable” state (where molecules strongly associate with proteins or starch) to a “freezable” state capable of forming ice crystals (Simmons, Smith, & Vodovotz, 2012). It was observed that the freezable water content of pre-fermented frozen dough significantly increased after 10 days of storage (*p* < 0.05) and continued to increase with longer storage time (Table 1). These changes proved that freezing weakened the water retention capacity of the These findings suggest that freezing weakens the water retention capacity of the pre-fermented dough. As frozen storage progresses, the moisture state becomes more fluid due to the growth of ice crystals. This change has the potential to disrupt hydrogen and ionic bonds, resulting in reduced gluten crosslinking and diminished ability to bind water. Consequently, some deeply bound water is converted into weakly bound water and free water (Fig. 1B). The increasing number and size of ice crystals (Fig. 1F) further contribute to the breakdown of disulfide bonds and reduction in polymer protein content (Wang, Yang, Gu, Xu, & Jin, 2017), this is consistent with the results of Yang ‘s study (Yang et al., 2022). These changes lead to the loss of semi-bound water and collapse of the gluten network. Additionally, subzero temperatures cause significant alterations in the physicochemical properties of the hydrated gluten network due to ice formation and recrystallization driven by isothermal or temperature fluctuations (Ding et al., 2020). Ultimately, these modifications affect the network structure of gluten, resulting in decreased dough water retention, freezing cracks on the surface, structural collapse, and cooking quality defects (Omedi, Huang, Zhang, Li, & Zheng, 2019).

In order to enhance the quality of pre-fermented frozen dough and address water migration during the freezing storage process, CF and SDF were introduced into the dough. The aim was to improve water distribution within the dough by leveraging their high-water retention properties. Both CF and SDF exhibited superior water holding capacity compared to wheat flour, as evident from their infrared absorption spectra and XRD characteristic diffraction peak (Fig. 4A B). Moreover, they played a significant role in enhancing moisture distribution in pre-fermented frozen dough during storage.

The addition of CF and SDF resulted in a reduction of freezable water content in pre-fermented frozen dough during storage. Notably, SDF exhibited a higher water holding capacity than CF, leading to substantially lower freezable water content in the SDF group compared to the CF group. Additionally, the SDF group demonstrated a higher content of deeply bound water than the CF group (Fig. 1). This implies that less water is available for ice crystal formation in pre-fermented frozen dough when SDF is added, thereby offering some protection to the gluten protein structure (Fig. 2). However, contrary to Fan's research (Fan et al., 2024) on the reduction of free sulfhydryl groups and the protection of protein secondary structure, we speculated that the content and types of fibre may be caused by the difference. Overall, incorporating CF and SDF into pre-fermented frozen dough can significantly improve water distribution within the dough, reduce the presence of freezable water capable of forming ice crystals, and mitigate damage caused by ice crystals to the gluten protein in the pre-fermented frozen dough.

Although fibre possesses water-retention properties, there are still some drawbacks that need to be addressed and improved in the dough. Firstly, the addition of fibre can dilute the gluten network structure, thereby reducing its ability to retain gases. Secondly, the tips of the fibres can puncture gas cells, resulting in compromised stability and uniformity of the gluten network structure (N. Liu et al., 2021). Furthermore, the inclusion of CF and SDF alters the dynamic rheological properties of the dough. This includes an increase in viscosity modulus and a restriction in the movement of freezable water molecules. Consequently, there is a decrease in the content of freezable water within the dough, leading to a stiffer dough. The enhanced mechanical strength eventually leads to an increase in the chewiness of the Shaobing. Therefore, when adding fibre to pre-fermented frozen dough, it becomes necessary to incorporate food additives with strong gluten effects to compensate for any potential damage caused by the fibre to the gluten structure. In addition, according to zhang's review (Zhang, Fan, Xu, & Xu, 2024), pre-leavened dough needs a proper freezing rate during freezing to better protect the dough from freezing damage.

## 5 Conclusion

During the frozen storage process, the content of freezable water and free water increased, resulting in the formation of large ice crystals in the dough, the destruction of gluten network, resulting in the change of protein secondary structure, the increase of irregular curling, the decrease of α-helix content, the disruption of gluten structure order, the increase of hardness of pre-fermented dough after frozen storage, and the decrease of G' and G". According to the infrared absorption spectra and XRD characteristic diffraction peaks of CF and SDF, both of them have better water holding capacity than single wheat flour. Therefore, the pre-fermented frozen dough with CF and SDF added shows less ice crystals, freezable water and free water that is easy to form ice crystals than the dough in the control group. Compared with CF and SDF, SDF has a higher water retention capacity, so it is better in protecting the water migration of pre-fermented frozen dough. However, since fibre can dilute the gluten network and disrupt its ability to hold gas, it is necessary to combine it with other food additives that have strong gluten effects.

## CRediT authorship contribution statement

**Tianyu Xiao:** Writing – original draft, Methodology, Investigation. **Mingkun Sun:** Software, Methodology. **Shuwang Cao:** Resources. **Jianxiong Hao:** Supervision. **Huan Rao:** Supervision, Project administration. **Dandan Zhao:** Writing – review & editing. **Xueqiang Liu:** Writing – review & editing.

## Declaration of competing interest

The authors declare that there are no conflicts of interest.

## Data Availability

No data was used for the research described in the article.

## Appendix A Supplementary data

Supplementary data 1 Supplementary material Image 1
