# Supplementary material for: Enhancing water retention and mechanisms of citrus and soya bean dietary fibres in pre-fermented frozen dough
Source: Food Chem X. 2024 Mar 5;22:101269. doi: 10.1016/j.fochx.2024.101269 (PMC10943030; doi:10.1016/j.fochx.2024.101269)
Supplement: Supplementary data 1 — Supplementary material [file mmc1.docx]

**Enhancing Water Retention and Mechanisms of Citrus and Soya Bean Dietary Fibers in Pre-Fermented Frozen Dough**

Tianyu Xiao^1^, Mingkun Sun^1^, Shuwang Cao^2^, Jianxiong Hao ^1,*^, Huan Rao ^1,*^, Dandan Zhao^1^ and Xueqiang Liu^1^

^1^ College of Food Science and Biology, Hebei University of Science and Technology, No.26 Yuxiang Street, Shijiazhuang, China

^2^ Shijiazhuang Beirong Foods Co., Zhengding, Shijiazhuang, China

**Supplementary Data**

**1 Materials and methods**

**1.1 Moisture distribution determination of pre-fermented frozen dough**

Carr-Purcell-Meiboom-Gill (CPMG) pulses were used for the LF-NMR measurements, conditions see Table s-1.

Table s-1 Operation conditions of LF-NMR

| SW（kHz） | TD | TW（ms） | TE（ms） | NECH | NS |
| --- | --- | --- | --- | --- | --- |
| 250 | 400020 | 2500 | 0.2 | 8000 | 4 |

**1.2 Nuclear magnetic imaging analysis of pre-fermented frozen dough**

The conditions for the operation of nuclear magnetic imaging are shown in Table s-2

Table s-2 Operation conditions of NMR

| MF (MHz) | SF (KHz) | TE（ms） | NECH | NS |
| --- | --- | --- | --- | --- |
| 21 | 250 | 0.2 | 8000 | 12 |

**1.3 Texture properties of pre-fermented frozen dough**

The TPA conditions for pre-fermented frozen dough can be found in Table s-3.

Table s-3 TPA conditions of pre-fermented frozen dough

| Test conditions | Number |
| --- | --- |
| Pre-test speed（mm/s） | 5.0 |
| Test speed（mm/s） | 5.0 |
| Post test speed（mm/s） | 5.0 |
| Compressibility（%） | 75.0 |
| Interval time（s） | 0.1 |
| Induction force（g） | 5.0 |

**1.4 Texture properties of raw Shaobing**

The TPA conditions of pre-fermented frozen raw Shaobing are referred to Table s-4.

Table s-4 TPA conditions of pre-fermented frozen raw shaobing

| Test conditions | Number |
| --- | --- |
| Pre-test speed（mm/s） | 1.0 |
| Test speed（mm/s） | 5.0 |
| Post test speed（mm/s） | 5.0 |
| Compression distance（mm） | 7.0 |
| Interval time（s） | 5.0 |
| Induction force（g） | 5.0 |
